# Brain activity during a working memory task after daily caffeine intake and caffeine withdrawal: a randomized double-blind placebo-controlled trial

**DOI:** 10.1038/s41598-022-26808-5

**Published:** 2023-01-18

**Authors:** Yu-Shiuan Lin, Janine Weibel, Hans-Peter Landolt, Francesco Santini, Helen Slawik, Stefan Borgwardt, Christian Cajochen, Carolin Franziska Reichert

**Affiliations:** 1 Centre for Chronobiology, University Psychiatric Clinics Basel, Wilhelm-Klein Strasse 27, 4002 Basel, Switzerland; 2 Transfaculty Research Platform Molecular and Cognitive Neurosciences, University of Basel, Basel, Switzerland; 3 Neuropsychiatry and Brain Imaging, Psychiatric Hospital of the University of Basel, Basel, Switzerland; 4 Institute of Pharmacology and Toxicology, University of Zurich, Zurich, Switzerland; 5 Sleep and Health Zurich, University Center of Competence, University of Zurich, Zurich, Switzerland; 6 Division of Radiological Physics, Department of Radiology, University Hospital Basel, Basel, Switzerland; 7 Department of Biomedical Engineering, University of Basel, Basel, Switzerland; 8 Clinical Sleep Laboratory, Psychiatric Hospital of the University of Basel, Basel, Switzerland

**Keywords:** Cognitive neuroscience, Human behaviour

## Abstract

*Acute* caffeine intake has been found to increase working memory (WM)-related brain activity in healthy adults without improving behavioral performances. The impact of *daily* caffeine intake—a ritual shared by 80% of the population worldwide—and of its discontinuation on working memory and its neural correlates remained unknown. In this double-blind, randomized, crossover study, we examined working memory functions in 20 young healthy non-smokers (age: 26.4 ± 4.0 years; body mass index: 22.7 ± 1.4 kg/m^2^; and habitual caffeine intake: 474.1 ± 107.5 mg/day) in a 10-day caffeine (150 mg × 3 times/day), a 10-day placebo (3 times/day), and a withdrawal condition (9-day caffeine followed by 1-day placebo). Throughout the 10th day of each condition, participants performed four times a working memory task (N-Back, comprising 3- and 0-back), and task-related blood-oxygen-level-dependent (BOLD) activity was measured in the last session with functional magnetic resonance imaging. Compared to placebo, participants showed a higher error rate and a longer reaction time in 3- against 0-back trials in the caffeine condition; also, in the withdrawal condition we observed a higher error rate compared to placebo. However, task-related BOLD activity, i.e., an increased attention network and decreased default mode network activity in 3- versus 0-back, did not show significant differences among three conditions. Interestingly, irrespective of 3- or 0-back, BOLD activity was reduced in the right hippocampus in the caffeine condition compared to placebo. Adding to the earlier evidence showing increasing cerebral metabolic demands for WM function after acute caffeine intake, our data suggest that such demands might be impeded over daily intake and therefore result in a worse performance. Finally, the reduced hippocampal activity may reflect caffeine-associated hippocampal grey matter plasticity reported in the previous analysis. The findings of this study reveal an adapted neurocognitive response to daily caffeine exposure and highlight the importance of classifying impacts of caffeine on clinical and healthy populations.

## Introduction

Caffeine is the most commonly consumed psychostimulant worldwide^1–3^. The *acute* benefit of caffeine intake on both simple and complex attention processes has been frequently reported^4–8^. Hence, it is tempting to examine the impact of *daily* caffeine on higher-order cognitive functions. The execution of working memory function relies on basic low-order cognitive processes such as attention and motor control^9,10^ and lies at the basis of several high-order cognitive functions. Given the psychostimulation of caffeine that enhances attention and motor responses, caffeine may also enhance the apparent performance in working memory tasks without actually influencing the true memory function^7^. Acute caffeine intake was frequently found to shorten the reaction time in working memory performance or improve the overall performance without dissociating the enhanced attention process by caffeine^11–14^. Interestingly, studies that separated or statistically controlled for caffeine effects on low-order task performance (i.e. using high- against low-workload task to control for low-order processes) often reported no clear-cut *net* benefits on working or short-term memory function^15–20^.

Among the studies controlling for low-order task performance, some of them also measured working memory-related blood-oxygen-level-dependent (BOLD) activity and consistently reported an acute increase in brain activity by caffeine, but no concomitant change in behavioral performance^17–19^. Koppelstaetter, et al.^17^ found that 100 mg caffeine acutely increased task-related BOLD activity in medial frontal regions without affecting the performance in the so-called N-back task, a classic working memory task that allows quantifying both low-order cognitive function and working memory with different workloads. Similarly, with 100 mg caffeine, Klaassen, et al.^18^ found an increased activity in the dorsolateral prefrontal cortex (DLPFC) in the encoding phase in a letter Sternberg task and a decreased thalamic activity in the maintenance phase. Again, there was no significant benefit of caffeine on behavioral performance but instead a worse accuracy in the high-workload trials. In addition, the task accuracy was associated with the load-related DLPFC activity. Similar to Koppelstaetter, et al.^17^, Haller, et al.^19^ found that 200 mg caffeine led to an increased task-related BOLD activity in widespread cortical and subcortical regions in older healthy individuals without a significant difference in the N-back performance. The same team later on compared the effect of 200 mg caffeine on working memory performance between cognitive-stable and cognitive-declined elderly populations. Again, different post-caffeine activation in the default mode network (DMN) between two groups was observed without significant behavioral effects^20^. Collectively, the evidence consistently suggests that the pharmacophysiological effects of caffeine could affect task-related brain functions without necessarily causing an apparent change in the behavioral performance of working memory tasks. Underlying the absence of behavioral changes and an increased task-related brain activity, it could be that caffeine increases demands for neural metabolic engagement to achieve the same level of performance as under placebo.

To date, it is unclear if such cerebral effects persist during daily exposure to caffeine, especially in a healthy cognitive state. Chronic administration of caffeine can lead to adaptions in the adenosine system, such as upregulated extracellular endogenous adenosine concentration^21^ and adenosine A1 receptors (A1R)^22,23^, thereby altering the balance between the counteractive A1 and A2A receptors (A2AR) signaling^24,25^. Behaviorally, chronic caffeine administration leads to tolerance to the psychostimulation of caffeine in rodents^26–28^. In the neurocognitive domain, however, the effects of chronic caffeine intake were majorly investigated in disease models, in which chronic caffeine administration was shown to rescue cognitive deficits induced by chronic stress, neurodegenerative diseases, and aging in rodents^29–32^. The normalizing effect of caffeine in these models were often based on the preconditions of an upregulated expression or function of adenosine A2AR in the striatum^33–35^ or hippocampus^36–39^ caused by the aforementioned disease or psychophysiological conditions. The preconditions therefore limited the extrapolation of the reported neuroprotective effects of daily or chronic caffeine intake to a healthy neural system. In humans, the effects of long-term caffeine intake on memory functions were mostly investigated by observational studies and have yielded mixed findings (discussed in^40^). Strictly controlled clinical studies examining effects of daily caffeine intake on memory functions remain scarce.

Hence, we aimed to use a double-blind randomized placebo-controlled crossover study to answer whether daily caffeine intake increased working memory-related brain activity and improved behavioral performance. We measured working memory by N-Back tasks repeatedly across 13 h after waking up in a caffeine, a withdrawal, and a placebo condition. We defined a “*net*” working memory function with the performance in high work-load trials (3-back) controlled for the basic attention and motor process (0-back). Furthermore, as daily intake of caffeine was found to lead to physiological adaptions^21,22^ which may mediate some of the caffeine withdrawal symptoms^23^, we also investigated working memory function and its cerebral correlates in the window of the peak caffeine withdrawal^41^, which occurs between 24 and 36 h after discontinuing daily caffeine intake^41^. The repeated measurements enabled capturing the general responses to caffeine intake and to caffeine withdrawal reliably across times. The fMRI session was scheduled at 5 h after the last caffeine or placebo intake in order to reduce the impacts of acute caffeine effects on brain activity patterns. Finally, as caffeine intake reduces global perfusion and can potentially affect BOLD signals, we statistically controlled for the confounding effect of global cerebral blood flow in the analysis.

## Methods

### Ethics and registration

This study was conducted at University Hospital of Basel, Basel city, Switzerland. The ethical approval of the current study was issued by the Ethics Committee Northwest/Central Switzerland (EKNZ). The study execution has adhered to the declaration of Helsinki, and all participants have provided their informed consent voluntarily in a written form. The detailed study protocol can be found on the ClinicalTrial,gov registration (NCT05409339, ***“****Influence of Caffeine Consumption on the Human Circadian System”, 06/05/2022*).

### Volunteers

Overall, twenty healthy male volunteers (age: 26.4 ± 4.0 years; body mass index: 22.7 ± 1.4 kg/m^2^; and self-report habitual caffeine intake: 474.1 ± 107.5 mg/day) completed the study. The detailed demographic feature was reported in^42^, and the inclusion and exclusion criteria are listed in the Supplement File. Due to a confined sample size, the study focused on a male-only population in order to maximize the signal-to-noise ratio for the true effects by reducing the variability of caffeine metabolism rendered by hormonal fluctuation^43,44^ and contraceptives^45,46^ in females. The power analysis was conducted based on the vigilance-related BOLD activity in response to the changes in homeostatic sleep pressure^47^. We excluded data measured during a caffeine condition from one participant due to incompliance with the treatment. We lost data measured during one placebo condition from another participant due to a malfunction of the fMRI scanner.

### Study protocol

**Figure 1:**
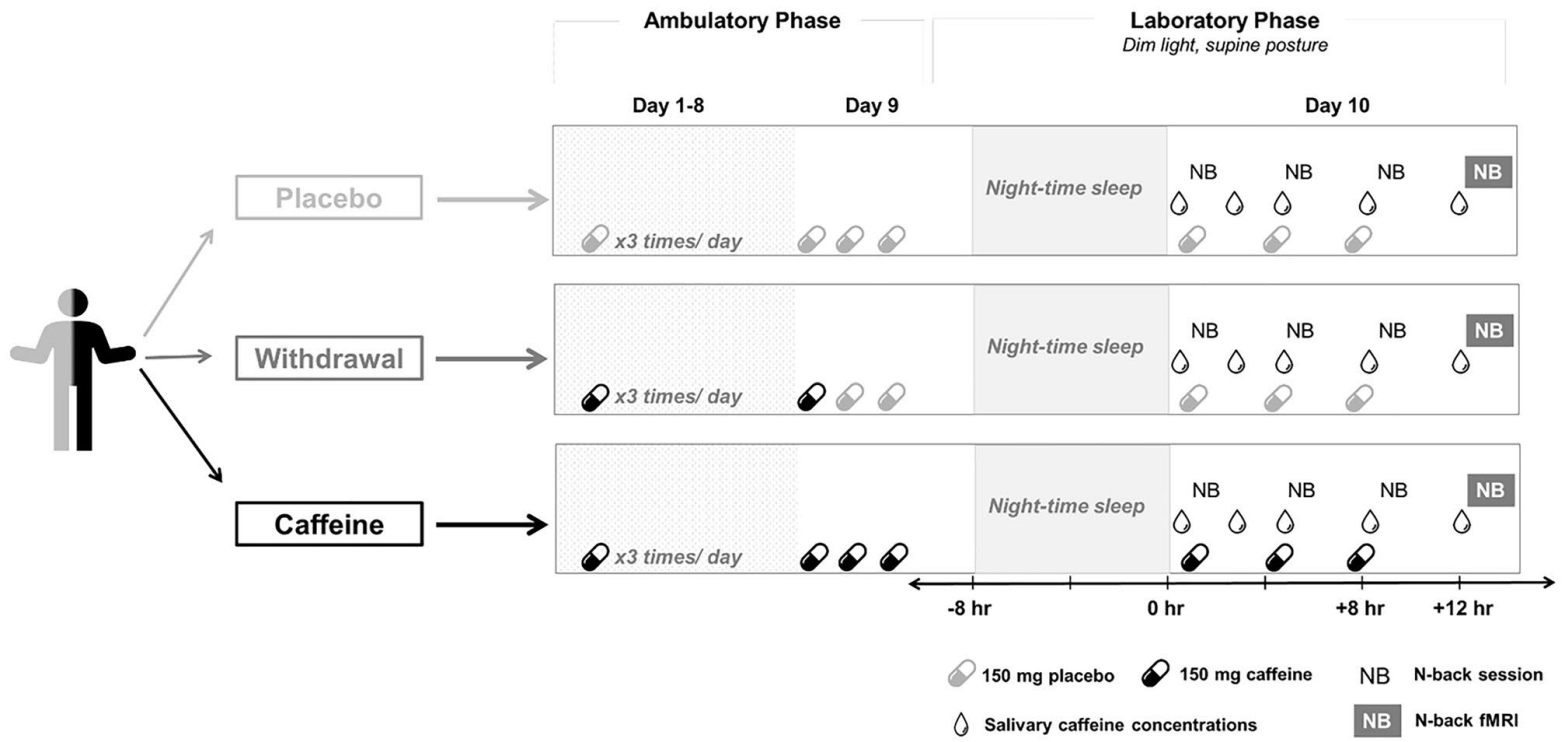
Study protocol.

In a double-blind, randomized placebo-controlled study, each of the 20 volunteers completed three conditions: placebo, caffeine, and caffeine withdrawal (Fig. 1). The order of the three conditions was randomized and semi-balanced, i.e. each order was assigned to a minimum of three participants, while *Withdrawal*—*Placebo*—*Caffeine* and *Placebo*—*Caffeine*—*Withdrawal* was assigned to four participants each. Participants were assigned sequentially according to an encrypted list of 20 randomized condition orders generated by a staff external to this study; the external staff was also in charge of allocating capsules for each study session. The duration of the data collection was from July 2016 until December 2018.

In each condition, volunteers underwent nine ambulatory days, followed by a 43-h laboratory stay which started in the evening of the ninth day. Instead of a washout period, we implemented the 9-day ambulatory placebo intake to ensure a clean state off from any remaining caffeine levels or withdrawal symptoms^41^. We also ensured a standardized daily administration of caffeine or placebo with a controlled dosage and times of intake. On each day, volunteers took treatments at around 45 min, 255 min, and 475 min after habitual rise time in the morning. In the placebo and the caffeine condition, the daily treatments throughout 10 days were 3 times mannitol or 3 times 150 mg caffeine plus additional mannitol, respectively; in the withdrawal condition, volunteers received caffeine capsules until the first intake of the 9th day, followed by placebo capsules as the second and third treatments until the end of 10th day. All capsules appeared identical, and the participants as well as all the staffs involved in recruitment, data collections, and data analysis were blinded to the conditions until the completion of the study. Participants’ rest-activity cycles were monitored by actimetry and kept constant between conditions in timing and duration (8-h sleep, 16-h wakefulness) within each individual.

Throughout each of the entire 10 days, volunteers abstained from caffeine-containing diets, including coffee, tea, energy drink, soda, and chocolate. The compliance was screened daily by collecting perspiratory caffeine levels in the evening [results see^42^]. During the laboratory stay, volunteers stayed in dim light (< 8 lx), constant half-supine position (∼45°), with controlled dietary and lavatory time. Water consumption ad libitum did not differ between conditions [results see^40^]. Volunteers were not allowed to use mobile phones and had no social contacts except with the study staffs.

Here, we focus on measurements (detailed in the following sections) acquired on the 10th day. All measurements were scheduled according to the individual’s habitual bedtime. The fMRI scan started at 13 h after habitual wake-up time (equivalent to 5 h after the last treatment). Participants performed visual working memory tasks (N-back) at 1 h, 5 h, 9 h, and 13 h (at scan) after awakening. We used salivary caffeine concentrations to ensure the successful administrations of the treatments in the laboratory.

No adverse events occurred during study execution.

### Measurements

#### N-back

We employed N-back tasks to assess visual working memory during the laboratory (10th) day of each condition. Four sessions were scheduled during the course of the day and allowed us to examine performances through and after caffeine intake. The timings of the four sessions relative to the treatments were: 15 min. after the first treatment, 1 h after the second, as well as 1 h and 5 h after the last treatment. Every session consisted of 9 trials of 3-back and 5 trials of 0-back, each trial consisted of 30 stimuli with 1.5 s interval in a quasi-random order. Each of the consecutively presented stimuli was presented for 1 s. Participants keyed down “1” when the presented stimulus (a letter) matched the one three stimuli earlier (3-back, “high workload”) or when it was a “K” (0-back, “low workload”), otherwise pressing “2”. Participants performed one practice session in the evening of the ninth day.

Performances were indexed by (a) error rate, calculated by the ratio of “incorrect rate” (missed + false alarms) to “correct response” (hits + correct rejections), and (b) reaction time (RT) during correct answers. We employed generalized linear mixed models to analyze the condition and time effects using R packages *afex*^48^ and *lme4*^49^. We defined “working memory function” by the proportion of the performance in 3-back against 0-back, as quantified by the error rate and RT in 3-back regressing out the variance of the error rate or RT in 0-back, respectively. Furthermore, we included orders of conditions as a covariate in order to control for learning effects. In our earlier report^40^, we conducted a preliminary analysis on the general performance averaged from all sessions on the 10th day of each condition. The current analyses were focused on a more thorough examination consisting of both the main effect of condition and the interaction effect between condition and different study sessions. Lastly, the task session taking place in the scanner was identical to all the other sessions except that the participants reacted with an fMRI-compatible response box. Participants were asked to react with index (for “1”) and middle finger (for “2”) irrespective of using a keyboard or a response box.

#### Functional magnetic resonance imaging (fMRI)

##### Acquisition

We used a two-dimensional multislice gradient-echo echo-planar imaging sequence (GRE-EPI, 3 × 3 × 3 mm^3^, TR = 2500 ms, TE = 30 ms, FA = 82°, number of slice = 39) to measure task-related BOLD activity, as well as a three-dimensional magnetization-prepared rapid gradient-echo (MP-RAGE) sequence (1 × 1x1mm3, TR = 2000 ms, TI = 1000 ms, TE = 3.37 ms, FA = 8°) to acquire T1-weighted structural images on a 3 T Siemens scanner (MAGNETOM Prisma; Siemens Healthineer, Erlangen, Germany).

##### Image preprocessing

We employed the preprocessing pipeline for EPI and T1-weighted images from CONN toolbox (http://www.nitrc.org/projects/conn) on MATLAB. The preprocessing of both structural and functional images started with the spatial realignment, unwarping, and corrections for slice-timing and motions (motion threshold set at 0.9 mm). Structural and functional images were normalized to a standard brain from Montreal Neurological Institute (i.e. MNI-space) independently, followed by the co-registration and the segmentation of grey matter (GM), white matter (WM), and cerebrospinal fluid (CSF). The functional and structural images were then resliced into 2 mm and 1 mm isotropic voxels, respectively. Finally, the functional images were smoothed with Gaussian kernel of 8 mm full width half maximum.

##### Classical functional analysis

The statistical analysis was performed on SPM12. In the first level analysis, we used 3- and 0-back as regressors of interests and six estimated parameters of motion as regressors of no interests. Signal drifts were adjusted by a high-pass filter of 128 s and serial correlations using an AR(1) model. We contrasted the activation by workloads, i.e., 3-back > 0-back, 3-back < 0-back, and load-independent responses. In the group-level analysis, we used a flexible factorial model to estimate condition effects as the fixed effect, subject effects as the random effect, and age as a covariate, in each workload contrast. In order to control for the caffeine- and withdrawal-induced changes in perfusion, we used individual whole-brain cerebral blood flow, measured by arterial spin labelling (sequence detail see the *Acquisition* section in methods of^40^), as a covariate of ANCOVA for global normalization. The statistically significant thresholds were set at a voxel-level uncorrected *p* < .001, and at a cluster-level threshold set at *p*_FWE_ < 0.05.

##### Functional connectivity analysis

The functional connectivity analysis was an exploratory step based on the observations in the cluster analysis. Hence, we employed a ROI-to-ROI approach and focused on the hippocampus and middle frontal gyri. The ROIs were defined and segmented based on the FSL Harvard–Oxford atlas (including 132 ROIs).

We performed a functional connectivity analysis using the CONN toolbox. Before the classical first level analysis, a denoising step using the anatomical component-based noise correction procedure (aCompCor) was implemented. We used linear regressions to remove the potential confounding effects in the BOLD signal, including the noise signals derived from WM and CSF, the estimated motions, the identified outlier scans (i.e. scrubbing), and the constant and first-order linear session effects. Further, we applied a temporal band-pass filter of 0.008–0.09 Hz to reduce the low-frequency drift derived from physiological sources or head-motions.

The first level analysis was performed with a weighted general linear model with bivariate correlations. We used a Hemodynamic Response Function (HRF) to weigh down the beginning for a delay of the BOLD response. For the group analysis, we used two workloads and three conditions as regressors of interest and age as regressor of no interest. The thresholds of statistical significance were set at a voxel-level uncorrected *p* < .001 and a cluster-level threshold at *p*_FWE_ < 0.05.

#### Salivary caffeine levels

We collected saliva samples in a 2-h interval from 15 min before the first treatment intake until the end of the laboratory day in each condition. As a validation for successful treatments, we focused on the five samples: before caffeine administration, 1 h after each administration, and 15 min before the scan. The salivary caffeine concentrations were quantified by a High-Performance Liquid Chromatography (HPLC) coupled to tandem mass spectrometry at the Laboratory Medicine, University Hospital Basel. The chromatographic separation was done by an analytical ion exchange phase column for methyl malonic acid. The detection threshold of the method was 20 ng/ml. We analyzed the saliva sampling points x condition effects on caffeine levels by generalized linear mixed models with R packages *afex*^48^ and *lme4*^49^.

## Results

### Caffeine levels

The salivary caffeine data confirmed a successful experimental manipulation in the placebo, caffeine, and withdrawal condition. A significant interaction effect between condition and saliva sampling points (F_2,91_ = 17.7, *p* < .001) indicated a significant increase of caffeine concentrations from the baseline to the time of scan in the caffeine condition, a significant decrease in concentration in the withdrawal condition, and no significant change in caffeine concentrations in the placebo condition. For data details, please find the supplementary results (Table S1).

### Cognitive performance

#### Basic attentional and psychomotor performance (0-back)

**Figure 2:**
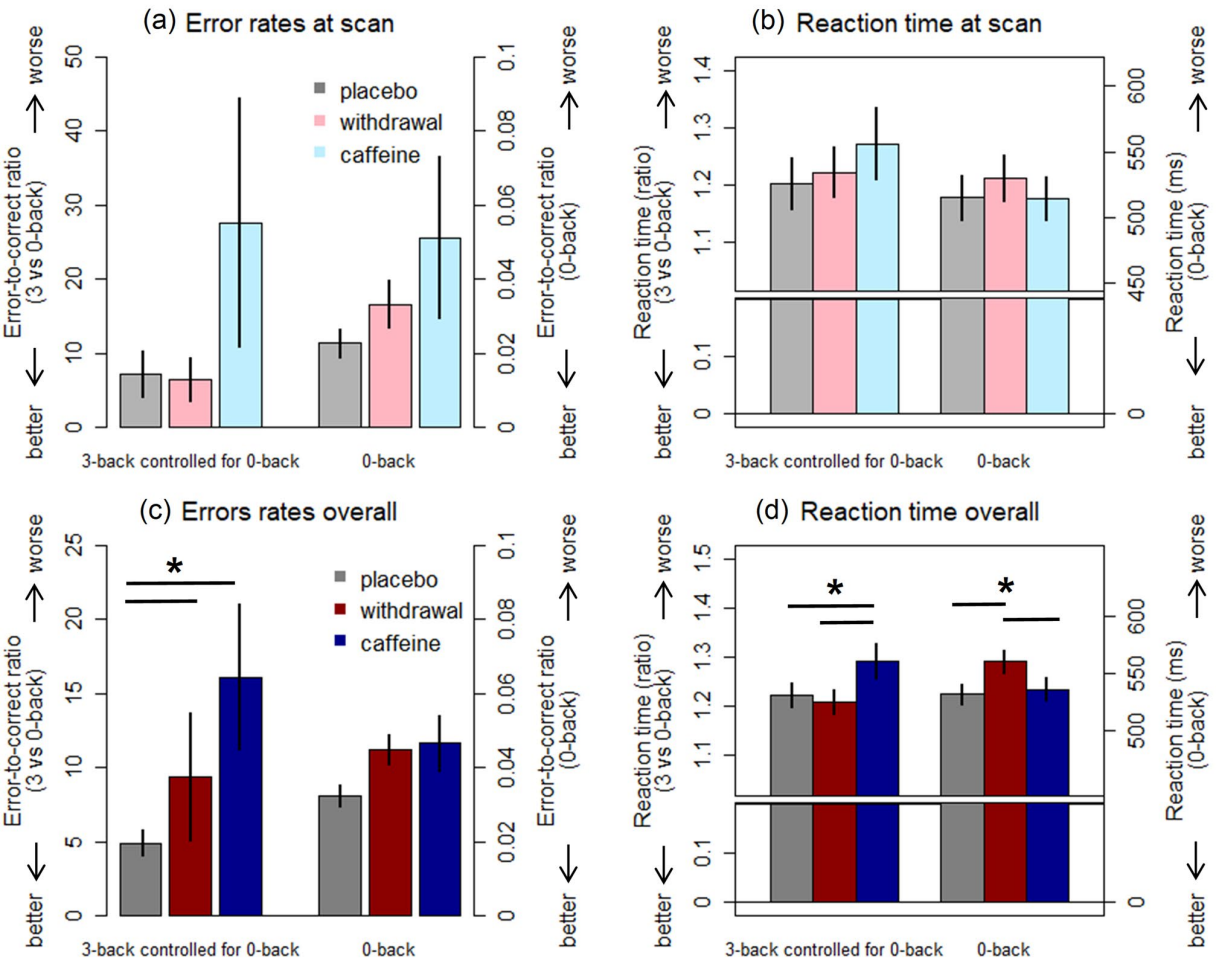
Main effect and time course of N-back performance per condition. **(a,b)** display the main effects of conditions at scan session with means and standard errors of the error rates (left panel) and the reaction time (RT, right panel). The three bars on the left side of each plot presents the performance in 3- proportional to 0-back, while the other three on the right side of each presents the performance in 0-back tasks. In an identical fashion, (**c**,**d**) display the main effects of conditions over all four sessions. We omit separated displays by sessions since the condition effects did not significantly differ between sessions (i.e. no significant interaction effects; please see "Result"section). However, we provide a separate panel for the performance at the scan session, i.e. (**a**,**b**), which allows integrating the behavioral information with the fMRI results. *Note:* in this visualization, we use the proportions of 3- to 0-back to adjust the performance of 3-back for 0-back. In statistics, we used raw data of 3-back as the outcome variables and regressed out the variance of 0-back in a linear mixed model.

On error rates in 0-back, we observed neither a significant effect of condition (F_2,198_ = 1.9, *p* = .152, Fig. 2) nor a significant condition x session interaction (F_6,198_ = 1.4, *p* = .216) [Median (quartile) of error rates in 0-back: placebo 0.02 (0.01 – 0.03); caffeine: 0.02 (0.01 – 0.04); withdrawal 0.03 (0.02 – 0.04)]. Independent of conditions, we found a significant session effect (F_3,198_ = 5.1, *p* = .002), for which a post-hoc analysis indicated a worse performance in Session 2 and 3 compared to both Session 1 (p_all_ < 0.040, Cohen’s d = 0.46 and 0.49) and scan session (p_all_ < 0.007, Cohen’s d = 0.57 and 0.59).

In the RTs in 0-back, we observed a significant effect of condition (F_2,198_ = 9.6, *p* < .001). A post-hoc analysis suggested a longer RT in the withdrawal condition compared to both the placebo (*p* < .001, Cohen’s d = 0.68) and the caffeine condition (*p* = .003, Cohen’s d = 0.58). No significant difference was found between caffeine and placebo in RTs (*p* = .815). [Mean ± SD of RTs (ms) in 0-back: placebo 531.7 ± 71.4; caffeine: 536.1 ± 85.6; withdrawal 560.3 ± 83.5]. Moreover, we found a significant session effect (F_3,198_ = 8.3, *p* < .001), for which the post-hoc analysis indicated exceptionally shorter RTs in the scan session compared to all other sessions (*p*_all_ < 0.011, Cohen’s d = − 0.61 to − 0.92). No significant interaction between condition and session was found (F_6,198_ = 0.5, *p* = .790).

#### Working memory function (3-back controlled for 0-back)

On the *net* error rates in 3-back controlled for 0-back, we observed a significant main effect of condition (F_2,197_ = 5.0, *p* = .008). A post-hoc analysis indicated a higher *net* error rate in the caffeine (*p* < .031, Cohen’s d = 0.40) and withdrawal (*p* = .013, Cohen’s d = 0.45) condition compared to placebo. In Fig. 2, we illustrate this effect by presenting the ratio of 3-back to 0-back per condition. [Median (quartile) in 3-back: placebo 0.05 (0.03–0.09); caffeine: 0.07 (0.03–0.14); withdrawal 0.07 (0.03–0.14)]. The analyses did not indicate a significant difference between caffeine and withdrawal conditions (*p* = .960). No significant main effect of session (F_3,195_ = 1.4, *p* = .244) nor condition x session interaction (F_6,195_ = 0.3, *p* = .936) was found.

Similarly, we also found a significant main effect of condition on *net* 3-back RTs controlled for the variance in the RTs in 0-back (F_2,196_ = 6.6, *p* = .002), in which a significantly longer *net* RT was observed in the caffeine condition compared to both placebo (*p* = .003, Cohen’s d = 0.57) and withdrawal (*p* = .023, Cohen’s d = 0.47). No clear-cut difference was found in *net* 3-back RTs between withdrawal and placebo. In addition, we observed a significant session effect (F_3,195_ = 3.4, *p* = .019), in which the prominent difference was a significantly longer RT in the first compared to the last sessions (*p* = .022, Cohen’s d = 0.60). We did not find a significant condition x session interaction (F_6,196_ = 0.2, *p* = .960) in the RTs. [Mean ± SD of RTs (ms) in 3-back: placebo 647.5 ± 128.2; caffeine: 688.9 ± 179.0; withdrawal 671.2 ± 123.6].

### Functional MRI analysis

**Figure 3:**
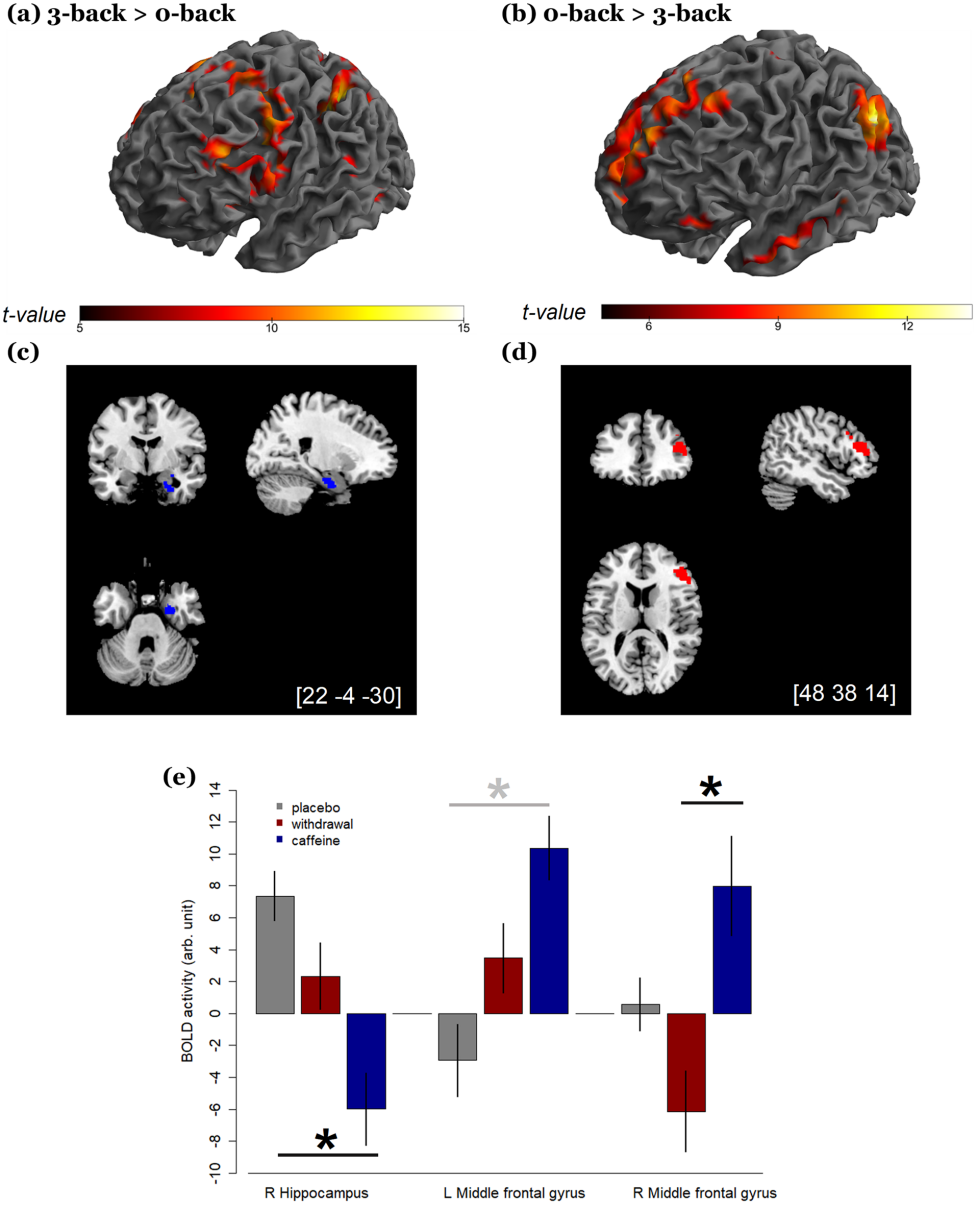
The effects of workload and conditions on blood-oxygen-level-dependent (BOLD) activity. (**a**) brain regions showing increased activities during 3-back compared to 0-back. **(b)** brain regions showing decreased activities during 3-back compared to 0-back. **(c)** blue color indicates the location of reduced right hippocampal BOLD activity (*p*_FWE_ < .05) during daily caffeine intake compared to placebo. **(d)** red color indicates the location of increased BOLD activity (*p*_FWE_ < .05) in the right middle frontal gyrus during daily caffeine intake compared to withdrawal. **(e)** Eigenvariates of the significant clusters in hippocampus and left and right middle frontal gyri. The black asterisks and black lines indicate the statistically significant contrast, while the grey asterisk and the grey line indicate the contrast only exhibited at trend differences.

The statistical results from the functional analysis are presented in detail in Table 1. Independent of conditions, the whole brain analysis indicated a significant difference in BOLD activity between 3- and 0-back (Fig. 3). In particular, we observed an increased activity in the bilateral attention and motor regions in 3-back compared to 0-back, including the premotor and supplementary motor areas, parietal lobes, and superior temporal gyrus (*p*_FWE_-all < 0.001). Moreover, we found a reduced activity in 3-back compared to 0-back in the DMN regions and parts of the limbic system, including left posterior cingulate cortex (*p*_FWE_ < 0.001), left medial prefrontal cortex (*p*_FWE_ < 0.001), bilateral precentral gyri (*p*_FWE_ < 0.002), bilateral angular gyri (*p*_FWE_ < 0.001), bilateral medial temporal gyri (*p*_FWE_ < 0.001), and bilateral hippocampi (*p*_FWE_ < 0.009). However, these load-dependent activities did not show a significant difference among the three conditions.

**Table 1:** The table presents the SPM results, including peak level t-values, cluster level *p*_FWE_ values, and cluster sizes (K_E_).

Contrast	Neuromorphometrics	BA	MNI coordinate			t	*p*_FWE_	K_E_
			x	y	z			
**Work-load related BOLD activity**								
3 > 0 back	Left superior parietal lobe	7	− 36	− 42	46	16.0	< .001	254,860
	L anterior insula	13	− 28	24	0	15.9		
	R middle frontal gyrus	6	32	4	56	14.0		
	L superior frontal gyrus	6	− 22	4	52	13.0		
	L middle frontal gyrus	6	− 28	4	56	12.8		
	L supramarginal gyrus	6	− 8	14	52	12.22		
	R superior frontal gyrus	6	20	6	58	11.9		
	R supramarginal gyrus	8	10	20	44	11.7		
	R superior parietal lobe	40	38	− 36	44	12.4	< .001	1616
	L medial temporal lobe	21	− 50	− 44	12	7.9	< .001	621
	L superior temporal gyrus	22	− 58	− 28	4	4.9		
3 < 0 back	L angular gyrus	39	− 50	− 62	32	17.0	< .001	1302
	L posterior cingulate gyrus	23	− 2	− 46	34	12.0	< .001	4010
	L medial frontal lobe	10	− 12	58	26	11.7	< .001	7323
	L medial temporal gyrus	20	− 54	− 4	− 34	9.4	< .001	1508
	R angular gyrus	39	52	− 58	34	9.0	< .001	856
	R cerebellum	–	30	− 82	− 32	9.0	< .001	644
	L cerebellum	–	− 34	− 80	− 36	8.4	< .001	398
	L hippocampus	–	− 26	− 20	− 18	8.0	< .001	621
	R medial temporal gyrus	21	62	− 8	− 24	7.6	< .001	1568
	R inferior frontal gyrus	47	50	38	− 14	5.8	.025	264
	R hippocampus	–	26	− 18	− 18	6.7	.009	338
	L precentral gyrus	6	− 26	− 18	72	6.0	.002	442
	R precentral gyrus	6	40	− 14	62	5.3	.002	463
Placebo > caffeine	R parahippocampus	36	22	− 4	− 30	5.1	.035	243
	R hippocampus	–	18	− 8	− 24	5.0		
		–	28	− 8	− 16	4.0		
Caffeine > placebo	L middle frontal gyrus	10	− 24	44	20	4.7	.057	210
		10	− 30	48	26	4.3		
Caffeine > withdrawal	R middle frontal gyrus	46	48	38	14	4.5	< .001	632
		9	40	34	18	4.4		
		9	36	16	28	4.4		
		9	38	32	22	4.3		
		9	38	32	28	4.3		
	R Broca operculum	44	40	22	22	4.1		
	L inferior frontal gyrus	46	54	30	14	4.0		

We denote MNI coordinates of each region and their corresponding atlas labels based on Neuromorphometrics and the Brodmann’s area (BA) from whole-brain analysis. Multiple brain regions covered in a large cluster (such as left SPL et al*.* in 3 > 0 back or right parahippocampus et al*.* in placebo > caffeine) are displayed with shared cluster level statistics.

Irrespective of workloads, we observed an overall lower BOLD activity in the caffeine condition in a right hippocampal region (*p*_FWE_ = 0.035, Cohen’s d = − 1.3, Fig. 3) and an at-trend (*p*_FWE_ = 0.057, Cohen’s d = 1.2) higher activity in the left middle frontal gyrus, compared to placebo. Moreover, compared to withdrawal, we also found a higher activity in the right middle frontal gyrus in the caffeine condition (*p*_FWE_ < .001, Cohen’s d = 1.1). We did not observe a significant difference in the regional activation between the withdrawal and the placebo conditions.

Based on the reduced hippocampal activity and the *at-trend* increased activation in the middle frontal gyrus, we hypothesized that caffeine might strengthen the anticorrelation between these two regions. We exploratorily examined the functional connectivity between the bilateral hippocampus and the middle frontal gyri. However, we did not find a significant difference in the hippocampus-middle frontal gyrus correlations among conditions, irrespective of workloads.

Our previous analysis^40^ on the same participants revealed a significant association between a larger reduction in the right hippocampal volume and a higher area under the curve of caffeine plus its main metabolite, paraxanthine (AUC-CAPX). Based on the similarity of a reduced hippocampal activity in the current analysis, we examined the association between the reduction of hippocampal BOLD activity and the AUC-CAPX during the 12 h between the pre-caffeine- and the scan time (methodology details see^50^). Similar to the volumetric changes reported previously, we found a larger reduction in the hippocampal BOLD activity associated with a higher AUC-CAPX_0-12 h_. We address the statistics and results in the Supplement (Fig. S1).

## Discussion

Here we investigated working memory performance and underlying cerebral correlates after 10 days of regular caffeine intake and after caffeine withdrawal, compared to a 10-day placebo condition. Our data suggest that daily moderate-dose caffeine intake leads to a compromised working memory function, which may remain reduced during acutely withdrawing from the daily intake. However, similar to earlier studies^17–19^, changes in behavioral performance did not appear commensurately or similar as in brain activity patterns, which did not show clear-cut differences among conditions. Considering that acute caffeine might increase the neural metabolic demand for the same performance as suggested by the earlier evidence^17–19^, we speculate that such a demand may not be fulfilled during daily intake and therefore lead to a worse behavioral outcome. In addition, the reaction time in 0-back in our data confirm that daily intake leads to a tolerance to caffeine in the classical psychostimulation, e.g. reducing reaction time. Caffeine withdrawal, on the other hand, leads to an impaired attention process. Finally, potentially reflecting the functional expression of the reduced right hippocampal grey matter volume previously reported^40^, our data further add that daily caffeine intake may inhibit the hippocampal activity during memory task performance. Compared with the neuroprotective evidence of chronic caffeine intake in disease models, our findings suggest that daily caffeine intake can lead to a divergent outcome a healthy central neural system.

### Caffeine and working memory: increase cerebral capacity or demands?

The performance in a N-back task involves multiple hierarchical cognitive processes which heavily depend on different workloads. 0-back, a simple recognition-response task, involves mostly attention, motor control, and inhibition processes without challenging the memory capacity; 2- or more back requires cognitive components recruited in 0-back as well as an increasing memory capacity and continuous information updating. Earlier similar fMRI studies examining acute effects of caffeine using working memory tasks with high versus low-workload contrasts reported an increased task-related activity in the medial frontal region^17^, DLPFC^18^, and parietal regions^19^ after acute caffeine intake without behavioral improvement or even with a worse outcome in the demanding workload trials (i.e. 6- vs 0-back in N-back tasks^18^). The evidence implicates that caffeine may not increase the brain activity as to enhancing the capacity but rather increasing the neural metabolic demands for the same level of behavioral performance. Our findings on a poorer working memory performance without a significant change in the task-related activity therefore might be reflecting a failure in fulfilling such increased demands, thereby decreasing performances. Furthermore, the intermediate level of working memory reduction in the withdrawal between caffeine and placebo conditions further indicates that the presence and the dissipation of caffeine may be the key contributors to the changes of working memory performance. Notably, since participants took only 9 days of caffeine before the discontinuation in the withdrawal condition, the less reduction in the working memory performance in the withdrawal relative to the caffeine condition can be either due to a mitigation from the caffeine effect or simply because of a shorter caffeine exposure. In any case, working memory function seems to be altered in close response to the caffeine exposure and its dissipation, which appears to be contrary to the basic attention process and motor control which express the typical adaptions in the adenosine signaling over daily caffeine intake and caffeine withdrawal (detailed in the next section).

### Potential implications of caffeine effects for adenosine neuromodulation

The absence of the classic psychostimulation of caffeine and the impaired attention in caffeine withdrawal, as indexed by the performance in 0-back, corroborate earlier results in a psychomotor vigilance task^42^ suggesting a tolerance to daily caffeine intake in the vigilance enhancement as well as an impairment in vigilance in caffeine withdrawal^42^. Such responses correspond to the cumulated evidence on an adapted adenosine system during chronic caffeine exposure. The inhibitory adenosine A1R and facilitatory A2AR modulate the striatal neurotransmission through the allosteric interactions in the G-protein coupled receptor heterodimers, such as A1-dopamine D1 receptor heterodimer, A2A-dopamine D2 receptor heterodimer, as well as A2A-D2-metabolic glutamate receptors 5 (mGluR5) heterotetramer^51–53^. Caffeine, by non-selectively blocking adenosine receptors, *acutely* facilitates dopamine and glutamate transmission^22,54–58^ and behaviorally reduces fatigue^13,59^ as well as enhances attention^8^. However, *daily* caffeine intake can instead lead to an adaption in the adenosinergic properties, specifically an upregulation in the A1R signaling (by increasing genetic expressions^27^, upregulating affinity for agonists^60–63^ and downregulating affinity for antagonists^23,26^), as well as an accumulation in the extracellular endogenous adenosine concentration^21^. Such adaptions not only attenuate the behavioral effects of caffeine as a tolerance effect^26–28^ but also result in an strengthened inhibitory adenosine signaling after withdrawing from caffeine. The increased adenosine signaling may underlie the poorer attention in caffeine withdrawal through the inhibited striatal dopamine and glutamate transmission^64–66^.

Furthermore, ample in vitro evidence shows that, in contrast to the propensity of A1R in developing tolerance to caffeine, A2AR properties and the behavioral effects of A2AR antagonism are rather resistant over daily caffeine intake^26,67–70^. As elaborated earlier, the responses of working memory and attention processes to daily caffeine intake and caffeine withdrawal seem to follow distinct patterns. It is possible that the changes in the attention process is in expression of the adapted adenosine properties underlying the behavioral tolerance to caffeine and an upregulated inhibitory signal in caffeine withdrawal, while the changes in working memory function is primarily in expression of the A2AR antagonism by caffeine and the reduced antagonism in caffeine withdrawal.

The resistance of A2AR antagonism may further explain the reduced hippocampal activity during daily caffeine intake. Caffeine intake was found to reduce hippocampal long-term potentiation (LTP), a marker of synaptic plasticity, in freely behaving rodents^71^ and LTP-like cortical excitation in healthy people^72^. It is believed that the inhibited LTP by caffeine is primarily mediated by the antagonism of A2AR^73–75^ through the A2AR-modulated glutamate transmission in hippocampus^76,77^. Adenosine A2AR in the hippocampus is found to control the presynaptic glutamate release^78^ and modulate the postsynaptic N-methyl-D-aspartate receptor (NMDAR) signaling through the co-localization between A2AR and mGluR5^79^. Behaviorally, chronic caffeine administration can lead to a cross-tolerance to the NMDAR antagonist, MK-801, in the MK-801-induced hyperlocomotion in rodents^80,81^. Hence, during daily caffeine exposure, the maintained reduced LTP by hippocampal A2AR antagonism might underlie the inhibited hippocampal activity as observed in the current study.

Notably, there is evidence in non-caffeine consumers that decaffeinated espresso can impede the increase in cortical excitability triggered by a transcranial alternating current stimulation in a placebo condition^82^. The evidence implicates that other biocompounds (e.g., chlorogenic acid, caffeic acid, trigonelline, kahweol, and cafestol^83^) contained in coffee may potentially contribute to a reduced cortical excitability. Indeed, the selection of non-caffeine consumers, who most likely bear a genetic trait in the A2AR encoding gene (ADORA2A) for an extreme sensitivity to caffeine^84–88^, may also be a critical factor enabling the minor amount of caffeine contained in the decaffeinated espresso to reduce cortical excitability. Nevertheless, our data acquired in regular caffeine consumers highlights that solely caffeine, when consuming daily, may also lead to an inhibited activity as well as structural plasticity^40^ in hippocampus.

The predominance of A2AR antagonism underlying the effects of daily caffeine intake might also serve as a neurobiological fundament of the divergent impacts of caffeine on healthy versus clinical populations. Chronic caffeine administration has been frequently reported to exert beneficial effects under conditions involving an A2AR overexpression, including Parkinson’s disease^34,89–91^, dementia^29^, Alzheimer’s disease^32^, aging^31^, chronic stress^30^, or sleep deprivation^92–94^. Overexpressed A2ARs are found to be associated with a working memory deficit^95^, impaired motor control^96^ and catalepsy^97^, as well as depression and anxiety^96^. Normalizing the A2AR signals with chronic administration of caffeine or selective A2AR antagonist therefore may lead to an amelioration of the behavioral alterations. However, a constant A2AR antagonism with a decreased counteraction from A1R antagonism due to the daily caffeine-induced adaptions may imbalance the originally healthy dopamine and glutamatergic transmission^22,24,54,98^, leading to compromised neurobehavioral functions. More studies are warranted to stratify different populations and identify those who may be benefited most from a regular use of caffeine.

### Dorsolateral prefrontal cortex and attention process during daily caffeine intake

We observed a reduced BOLD activity in the medial frontal gyrus (encompassed by dorsolateral prefrontal cortex, DLPFC) in the withdrawal condition compared to caffeine. The DLPFC is critical for the execution of attention procesess^99,100^. Yet, daily caffeine intake elevates the neural engagement in DLPFC without improving attention performance, which is strikingly similar to the earlier evidence on the increased task-related brain activity with an absence of working memory improvement^17–19^. Furthermore, the reduced DLPFC activity in the withdrawal condition supports the observed poorer attention performance. Neocortex, including DLPFC, is predominantly abundant with A1Rs^101^ and scarcely expresses A2Rs^102,103^. Therefore, in DLPFC, the physiological expressions of adapted A1R signaling in response to daily caffeine exposure may prevail. As elaborated in the earlier paragraph, A1R signaling is prone to be upregulated over the caffeine exposure, leading to a behavioral tolerance to caffeine and a strengthened inhibitory adenosine signal after caffeine cessation. These characteristics of A1R-mediated neurobehavioral effects may explain the elevated DLPFC activity without an improved attention process in the caffeine condition as well as the reduced DLPFC activity and the poorer attention process in the withdrawal condition.

To summarize in a perspective of the hierarchical cognitive functions, caffeine intake in general seems to gain difficulty in cognitive engagement: at a behavioral level, pure attentional processing can be enhanced by acute caffeine^8^ but not by daily caffeine except for increasing the associated neural activity. Working memory performance, on the other hand, cannot be enhanced by acute caffeine intake except for increasing the associated neural activity^17–19^, furthermore, it is hindered by daily caffeine intake with no effects on associated neuroactivity. More studies are warranted to explore the molecular mechanism underlying the caffeine effects on different levels of cognitive functions as well as the dissociated neural and behavioral outcomes.

### Limitations and significances

A few limitations in our study should be considered when interpreting our data. First, caffeine can induce neurovascular uncoupling by reducing baseline CBF and increasing baseline cerebral metabolic rate of oxygen consumption (CMRO2)^104^. Although we mitigated the deviation by statistically adjusting for the variances of resting cerebral blood flow, if one were to resolve the issue more precisely, a simultaneous acquisition of CBF and BOLD activities would be recommended^104^. Second, despite a crossover design, the results might still be restricted by a relatively small sample size. In addition, due to a confined sample size, we limited the investigation on a male-only population in order to reduce the variability in caffeine metabolism derived from hormonal fluctuations^43,44^ and contraceptives^45,46^ in females. The minimization of variances may yield a better power for true pharmacological effects, but it is also at the expense of a good generalizability of the findings. Since we also found an association between the individual caffeine metabolism, as indexed by the AUC of caffeine and paraxanthine, and hippocampal activity, it is important to note that the observed caffeine effects in the current study might vary among females exhibiting different metabolism of caffeine or its metabolites due to influence of the estrus cycle^43,44^ or hormonal contraceptives^46^. Lastly, it is of importance to be aware that the observed effects in the current study were yielded from pure caffeine administration. The common caffeinated dietary, e.g., coffee or tea, contains several other biocompounds (such as chlorogenic acid, caffeic acid, trigonelline, kahweol, and cafestol^83^) which may provide other neuroprotective effects. Thus, one should not exclude a potential beneficial effect which caffeinated dietary may bring.

In conclusion, daily moderate-dose caffeine intake might lead to a compromised working memory performance, which remains reduced after withdrawing caffeine for 36 h. The impaired behavioral performance, together with the absence of changes in the task-related neural activity, suggest that the increased neural metabolic demand for working memory execution by acute caffeine^17–19^ may be impeded over daily intake. Furthermore, daily caffeine intake, potentially through a maintained A2AR antagonism, may inhibit hippocampal activity, which is implicated as a functional consequence of the hippocampal grey matter plasticity in our earlier report^40^. Nevertheless, the crossover design in the current study also suggests that the caffeine-associated responses may be restorable within 10 days of abstinence. Taken together, our findings, comparing to earlier evidence, reveal that the impacts of daily caffeine intake in young healthy adults might be divergent from an acute intake or from a deficient or pathological neural system. The divergency warrants more systematic investigations on the caffeine effects on different adenosine properties and functions in a stratified study population in order to provide precise recommendations on the caffeine use as a neuroprotective agent.

## Supplementary Information

Supplementary Information.

## Data Availability

All the data reported in this manuscript is available for research purpose upon requests. Please contact Lin.YuShiuan16@gmail.com for the request.
