# Supplementary material for: Brain activity during a working memory task after daily caffeine intake and caffeine withdrawal: a randomized double-blind placebo-controlled trial
Source: Sci Rep. 2023 Jan 18;13:1002. doi: 10.1038/s41598-022-26808-5 (PMC9849460; doi:10.1038/s41598-022-26808-5)
Supplement: Supplementary file 1 — Supplementary Information. [file 41598_2022_26808_MOESM1_ESM.docx]

**Supplement**

**Supplementary methods**

Inclusion, exclusion criteria

applicants aged between 18 and 35 years, BMI ≥ 18 and ≤ 25

non-shift workers, and without a history of transmeridian travels <1 month prior to study, were screened according to the following exclusion criteria:

- self-reported caffeine intake <300 or >600 mg/day (calculations were based on Bühler, et al. ^1^, adapted according to a classification of Snel and Lorist ^2^) to ensure the safety of caffeine intake and to exclude extreme response,
- bad sleep quality, i.e. PSQI> 5 in the last four weeks assessed by the Pittsburg Sleep Quality Index (PSQI) to control for sleep disturbances
- extreme chronotype, as defined by Horne-Ostberg's Morningness-Eveningness Score (HOMES) ≤ 30 or ≥ 70 to prevent pronounced variance in circadian phase.
- self-reported regular substance use (including medication, nicotine, and drugs) and other major medical conditions.

A habituation night in the laboratory was conducted to exclude poor sleep efficiency (SE < 70%) and clinical sleep disturbances (apnea index > 10, periodic leg movements > 15/h). A toxicological screening right before each laboratory session served to exclude the influence of recent drug intake including cannabis, amphetamine, methamphetamine, cocaine, benzocaine and morphine.

General physical health confirmed by laboratory physicians.

**Supplementary results**

Salivary caffeine concentration in each condition

| **Table S1. Salivary caffeine concentration (µg/ml)** **per condition.** Mean and standard deviation of the samples collected before the first treatment as well as before the fMRI scan session are presented per condition. “Baseline” indicates the first sample in the morning of the laboratory (10^th^) day before administration of scheduled caffeine or placebo. The asterisks indicate the samples showing significant increase from baseline to the time of scan compared to placebo. | | | | |
| --- | --- | --- | --- | --- |
|  |  | Placebo | Caffeine | Withdrawal |
| Baseline | | 0.03 ± 0.03 | 1.18 ± 1.24 | 0.46 ± 0.82 |
| Time of scan | | 0.03 ± 0.04 | 3.67 ± 2.08 *** | 0.12 ± 0.18 ** |

**: p < .01; ***: p < .001

Association between larger reduction in hippocampal BOLD activity and higher accumulation of salivary caffeine and paraxanthine concentration.

| 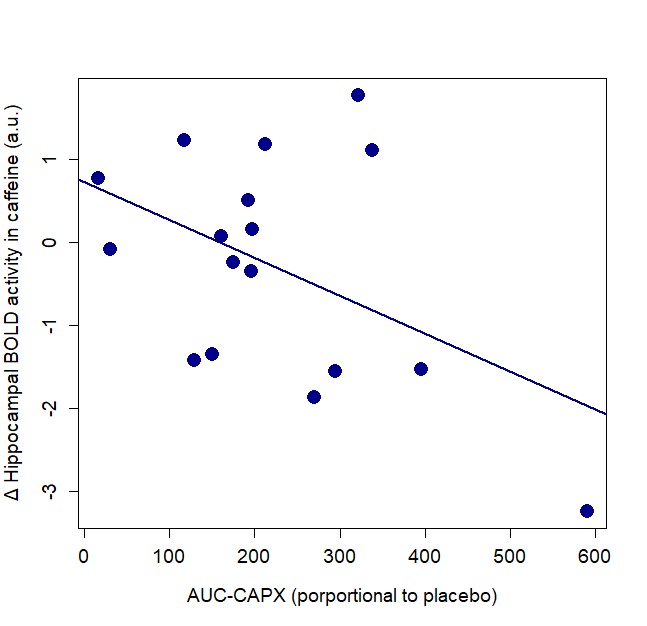 | **Fig S1. Supplementary analysis on the reduction of the hippocampal BOLD activity and the area under the curve of caffeine and paraxanthine (AUC-CAPX).** We conducted a regression analysis on the change in hippocampal BOLD response (ratio between caffeine and placebo) and the AUC-CAPX in caffeine proportional to placebo. The regression model indicated a small effect of AUC-CAPX (coefficient = -0.005, standard error = 0.002, t = -2.0). We used nonparametric test (“np” R package ^3^) in order to cope with the potential under-power issue due to a reduced sampling dimension and sample size. The significant test with a number of bootstrapping set at 5000 indicated a p-value of 0.048. The statistics suggested an association between a larger reduction in the hippocampal BOLD activity and a higher accumulation of caffeine + paraxanthine. Despite a relatively low sample size and larger variance, the alterations and their associations with AUC-CAPX are strikingly corresponding between the right hippocampal activity and volume. |
| --- | --- |

**References (for supplements)**

1 Bühler, E., Lachenmeier, D. W., Schlegel, K. & Winkler, G. Development of a tool to assess the caffeine intake among teenagers and young adults. *Science and Research* **61**, 58-63, doi: 10.4455/eu.2014.011 (2013).

2 Snel, J. & Lorist, M. M. Effects of caffeine on sleep and cognition. *Progress in brain research* **190**, 105-117, doi:10.1016/b978-0-444-53817-8.00006-2 (2011).

3 Hayfield, T. & Racine, J. S. Nonparametric Econometrics: The np Package. *2008* **27**, 32, doi:10.18637/jss.v027.i05 (2008).
